# Potential of *Cassia alata* L. Coupled with Biochar for Heavy Metal Stabilization in Multi-Metal Mine Tailings

**DOI:** 10.3390/ijerph15030494

**Published:** 2018-03-12

**Authors:** Lige Huang, Yuanyuan Li, Man Zhao, Yuanqing Chao, Rongliang Qiu, Yanhua Yang, Shizhong Wang

**Affiliations:** 1 School of Environmental Science and Engineering, Sun Yat-sen University, Guangzhou 510275, China; huanglig@mail2.sysu.edu.cn (L.H.); liyy97@mail2.sysu.edu.cn (Y.L.); zhaom26@mail2.sysu.edu.cn (M.Z.); chaoyuanq@mail.sysu.edu.cn (Y.C.); eesqrl@mail.sysu.edu.cn (R.Q.); yangyh6@mail.sysu.edu.cn (Y.Y.); 2 Guangdong Provincial Key Lab of Environmental Pollution Control and Remediation Technology, Guangzhou 510275, China; 3 Guangdong Provincial Engineering Research Center for Heavy Metal Contaminated Soil Remediation, Guangzhou 510275, China

**Keywords:** multi-metal mine tailings, biochar, *Cassia alata* L., ecological restoration

## Abstract

To explore the effect of different biochars on *Cassia alata* L. growth and heavy metal immobilization in multi-metal mine tailings, a 100-day pot experiment was conducted. Three biochars derived from *Hibiscus cannabinus* core (HB), sewage sludge (SB) and chicken manure (MB), were added to mine tailings at rates of 0.4%, 1% and 3% (*w*/*w*). The results showed that the root biomass, shoot biomass, plant height and root length were 1.2–2.8, 1.7–3.2, 1–1.5 and 1.6–3.3 times of those in the control group, respectively. Pb, Zn, Cu, Cd and As contents in the shoot decreased by 63.9–89.5%, 46.9–66.0%, 32.7–62.4%, 40.4–76.4% and 54.9–77.5%, respectively. The biochar significantly increased the pH and decreased the mild acid-soluble Pb and Cu concentrations in the mine tailings. Specifically, SB immobilized Pb and Cu better than MB and HB did, although it did not immobilize As, Zn or Cd. Meanwhile, more attention should be paid to the potential As release as the biochar application rate increases. In conclusion, *Cassia alata* L. coupled with 3% of SB could be an effective measure for restoring multi-metal mine tailings. This study herein provided a promising ecological restoration technique for future practice of heavy metal stabilization in mine tailings.

## 1. Introduction

China possesses diversified and large-scale mineral resources, accounting for more than 12% of the world’s proven reserves of mineral resources in 2010 [1]. Moreover, there are many types of polymetallic minerals with multi-metal paragenetic metallic minerals, including Pb, Zn, Cu, Cd, and as [2]. With the steady development of excessive metal mining and processing activities, mining and ecological environmental issues are giving rise to growing concern from the community, particularly with respect to mine tailings. The major industrial solid waste comprehensive utilization of the 12th Five-Year Plan of China indicates that mining activities generated 1.21 × 10^8^ tons of mine tailings, with less than 14% comprehensive utilization rate every year since 2010. Many tailings are still being dumped, forming tailing ponds. Mine tailings are the materials remaining after the extraction and beneficiation of ores, which contain an abundance of prominent toxic elements, such as Pb, Zn, Cu, Cd, and As [3]. These toxic elements can spread to surrounding areas through water erosion and wind erosion, which might cause water pollution and soil contamination and consequently pose risks to human health. Therefore, management and remediation of these mine tailings has become an important national issue concerning the national economy and people’s livelihood.

Phytostabilization is an effective and inexpensive rehabilitation strategy coupling metal-tolerant plants and mineral or organic soil amendments that help absorb and accumulate heavy metals in roots and promote precipitation within the rhizosphere [4], particularly for highly contaminated soils such as those containing mine tailings, which cannot be remediated by phytoextraction [5]. To date, phytostabilization research on multi-metal mine tailings is insufficient due to the extreme conditions posed by mine tailings, such as nutrient deficiencies, deficient inorganic matter and multi-metal toxicity. Thus, to provide an appropriate method for phytostabilization under such extreme mine tailing conditions, amendments should be considered to improve multi-metal mine tailing quality and stabilize contaminants, making conditions suitable for establishing metal-tolerant plants.

Amending with biochar has been shown to be an effective method of improving multi-metal-contaminated soil quality. Biochar is a carbon-rich co-product produced from biomass by pyrolysis under high-temperature and low-oxygen conditions. Biochar can be used as a soil amendment to improve soil quality and supply and retain nutrients, thereby enhancing plant growth [6,7,8]. Furthermore, biochar can absorb heavy metals to alleviate the associated toxicity toward plants through bulk surface area, pore size distribution, surface negative charges, high charge density and abundant functional groups [9,10,11]. Fellet et al. [12,13] also proposed that biochar can ameliorate substrates in terms of nutrient supply, decrease the availability of heavy metals in mine tailings and promote plant establishment, highlighting the potential of biochar as a soil amendment for multi-metal mine tailings. However, biochar’s effects in phytostabilization vary with biochar feedstock, biochar addition rate and the characteristics of the mine tailings. Further experiments should be performed to select the best biochar and biochar application rate for specific multi-metal mine tailings.

Metal-tolerant plants are another key factor for the ecological restoration of multi-metal mine tailings. *Cassia alata* L. is commonly known as a member of the *Leguminosae* family, an evergreen perennial shrub as well as a potential ornamental and medicinal plant that grows in the subtropical climate regions of China [14]. The plant’s medicinal functions in alleviating the symptoms of asthma attacks and ecological functions in enhancing resistance to soil ecosystem disturbances are becoming increasingly attractive to researchers [15]. However, studies have shown that *Cassia alata* L. cultivation might help reclaim degraded mining lands [16]. Practices involving the remediation of multi-metal-contaminated soil using *Cassia alata* L. have shown that the plant offers potential metal tolerance with good growth performance. However, the potential of this plant for the phytostabilization of multi-metal mine tailings has rarely been studied. Thus, in this study, a pot experiment was conducted to explore the effects of different biochars on *Cassia alata* L. growth and heavy metal immobilization in multi-metal mine tailings. This study is the first to examine the effects of *Cassia alata* L. coupled with different biochars and addition rates on heavy metal stabilization of multi-metal mine tailings.

## 2. Materials and Methods

### 2.1. Mine Tailings and Biochar

The mine tailings were collected from a Pb/Zn tailing pond in Meizhou, China (116°13′0″ E, 24°23′10″ N). The climate of the mine tailings pond is subtropical, with precipitation reaching 1473 mm per year and with an annual average temperature of 21.7 °C. The average relative humidity is 78%. The mine tailings pond, measuring 36 m in height, has accumulated 3.8 × 10^5^ m^3^ of mine tailings and is considered the fourth level tailing pond in China. The mine tailings were air-dried at room temperature and sifted through a 20-mesh sieve for the pot experiment.

The biochars were produced from kenaf (*Hibiscus cannabinus*) core, sewage sludge and chicken manure at 500 °C for 3 h, using the slow pyrolysis method described by Fellet et al. [12]. The biochars were named *Hibiscus cannabinus* biochar (HB), sewage sludge biochar (SB), chicken manure biochar (MB). The biochars were passed through a 20-mesh sieve for the pot experiment.

The basic properties of the mine tailings and biochars are presented in Table 1. The mine tailings had high total Pb, Zn, Cu, Cd and As contents (3642.7, 981, 70.5, 31.8 and 1587.1 mg·kg^−1^, respectively), a pH of 6.5, and low total carbon (TC) (0.3%) and total nitrogen (TN) contents (0.01%). The three biochars were suitable for alkaline soil amendment, with high TC and TN contents 23–250 and 39–275 times higher than those of the mine tailings, respectively. The biochars also had low heavy metal contents, except for SB, which showed high Zn (813.4 mg·kg^−1^) and Cd contents (2.6 mg·kg^−1^). BET surface area, Langmuir surface and pore volume of HB (117.61 m^2^·g^−1^, 232.63 m^2^·g^−1^ and 0.024 cm³·g^−1^) were higher than SB (14.10 m^2^·g^−1^, 71.46 m^2^·g^−1^ and −0.00073 cm³·g^−1^) and MB (6.52 m^2^·g^−1^, 20.74 m^2^·g^−1^ and −0.0013 cm³·g^−1^).

### 2.2. Pot Experimental Design

A pot experiment was conducted over 100 days under greenhouse conditions with the following 10 treatments (Table 2). The control group contained only 1 kg mine tailings (CK), while the amendment group featured three types of biochar amendment at rates of 0.4%, 1%, and 3% *w*/*w*. The rates of biochar were set in an appropriate range according to related works [17]. Specifically, the 1 kg mine tailings was mixed with the appropriate amounts of biochar; the mixture was then placed in a plastic pot (height of 11 cm, top diameter of 15 cm, and bottom diameter of 10 cm). All samples in the mine tailings group, which showed a 70% field water retention capacity, were incubated at 20–25 °C in the greenhouse for 14 days. 

Seeds of *Cassia alata* L., sterilized with 1% H_2_O_2_, were sown on top of silicon sand, and 7 days later, the seedlings were replanted in incubated tailings. All pots, with two plants per pot, were watered every other day under greenhouse conditions with natural light and at an ambient temperature of 20–25 °C. The plants were harvested for measurements 100 days after transplanting.

### 2.3. Sampling and Analysis

In the harvest stage, plants and soil samples were collected, and plant height, biomass and root length were measured by WinRHIZO (Pro STD4800, Regent Instruments Inc., Sainte Foy, QC, Canada). Each *Cassia alata* L. plant was divided into roots and shoots and rinsed with deionized water, dried at 105 °C for 1 h, stored at 60 °C for 3 days to allow the samples to reach a constant weight, and then ground into powders. All powders were digested using an acid mixture (HClO_4_:HNO_3_ = 1:4, *v*/*v*) at 180 °C on a hotplate and then determined by ICP-OES (Optima 5300DV, Perkin Elmer, Waltham, MA, USA).

The rhizosphere soil of each *Cassia alata* L. plant was air-dried and sequentially ground and passed through 20-mesh and 100-mesh sieves. Samples of 20-mesh soil were used to analyze the following parameters: pH, which was measured in a solid/water slurry (solid:water = 1:2.5 *w*/*w*); and NH_4_NO_3_-extractable metal content, using the method described by Gryschko et al. [18]. Samples of 100-mesh soil were used to analyze the following parameters: total heavy metals, digested using an acid mixture (HCl:HNO_3_:HClO_4_ = 3:1:1, *v*/*v*) at 180 °C on a hotplate and then determined by ICP-OES; C, H, N and S contents, using an elemental analyzer (Vario EL cube, Elementar, Langenselbold, Germany); and chemical fractionation of metals in soil, using the improved BCR sequential extraction method of the China National Standard (GB/T 25282-2010) [19].

### 2.4. Statistical Analysis

The experimental data were subjected to a one-way ANOVA and Duncan’s multiple comparison test (*p* < 0.05) with the SPSS statistical software package ver. 20 (SPSS Inc., Chicago, IL, USA).

## 3. Results

### 3.1. Effects of Different Biochar Treatments on Plant Growth

**Figure 1:**
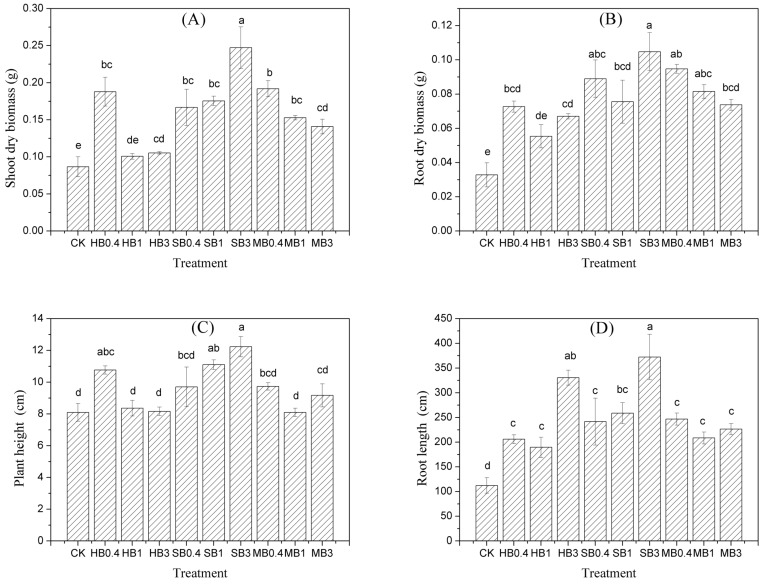
The effects of HB, SB and MB treatments on the plant growth. Shown are the (**A**) shoot biomass, (**B**) root biomass, (**C**) plant height and (**D**) root length of *Cassia alata* L. grown in mine tailings with different treatments. The vertical bars in the figures represent the standard errors of the means (*n* = 3), Different letters indicate significant differences among different treatments at a significance level of 0.05. CK, HB/SB/MB0.4, HB/SB/MB1 and HB/SB/MB3 refer to no biochar input and the incorporation of biochar into the soil at 0.4%, 1% and 3% by mass, respectively. HB: *Hibiscus cannabinus* core biochar; SB: sewage sludge biochar; MB: chicken manure biochar.

In general, the results (Figure 1) indicated that *Cassia alata* L. is a potential metal-tolerant plant because it was able to grow on multi-metal mine tailings without any amendments, and the amendments with different biochars had significant effects by increasing the root and shoot biomass and the height and root length of the plants. In contrast to the control group, the different biochar treatments remarkably increased the shoot biomass by factors of 1.2–2.8, except for the HB1 and HB3 treatments. Plants treated with SB presented the highest shoot biomass, and the shoot biomass of plants increased with the amount of added SB. Similarly, the root biomass was enhanced by the biochar treatments, reaching 1.7–3.2 times the biomass of the CK-treated plants, and only the HB1 treatment had no significant effect.

The data pertaining to plant height and root length indicate that the SB3 treatment provided outstanding acceleration in growth. The plant height and root length of the CK-treated plants were 8.1 cm and 112.4 cm, respectively, and the SB3 treatment increased plant height and root length to 12.1 cm and 370.9 cm, respectively. SB3 (1.5 times), HB0.4 (1.3 times) and MB0.4 (1.2 times) were the optimum treatments for increasing plant height. With respect to root length, the optimum addition rates were provided by SB3 (3.3 times), HB3 (2.9 times) and MB0.4 (2.2 times).

### 3.2. Heavy Metal Concentration in Shoots and Roots of Cassia alata L.

**Figure 2:**
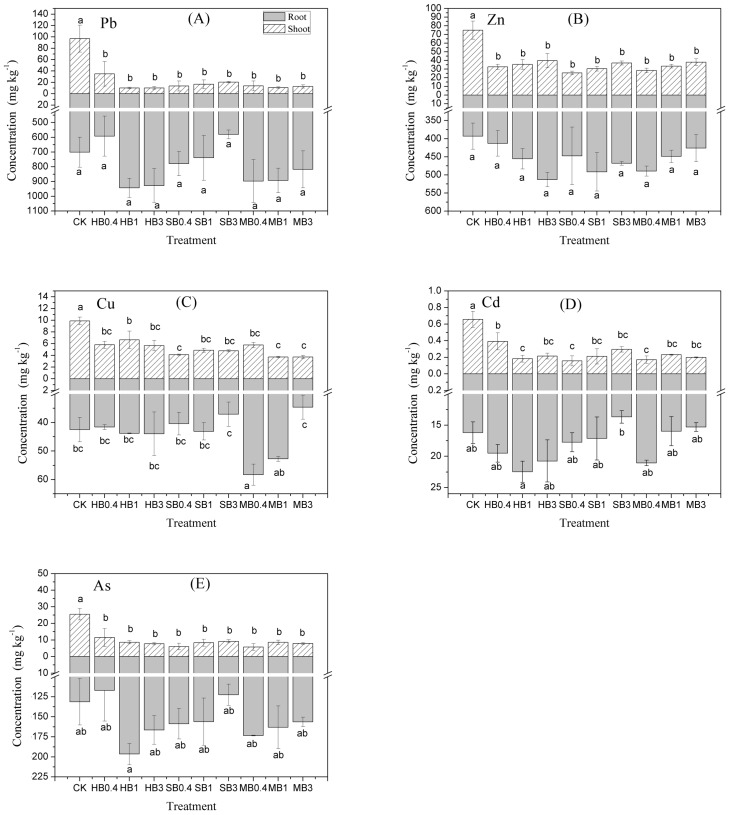
Effects of HB, SB and MB treatments on (**A**) Pb, (**B**) Zn, (**C**) Cu, (**D**) Cd and (**E**) As contents in shoots and roots of *Cassia alata* L. The vertical bars in the figures represent the standard errors of the means (*n* = 3), Different letters indicate significant differences among different treatments at a significance level of 0.05. CK, HB/SB/MB0.4, HB/SB/MB1 and HB/SB/MB3 refer to no biochar input and the incorporation of biochar into the soil at 0.4%, 1% and 3% by mass, respectively. HB: *Hibiscus cannabinus* core biochar; SB: sewage sludge biochar; MB: chicken manure biochar.

The effects of the different biochar treatments on the concentration of metals in the shoots and roots of *Cassia alata* L. plants are illustrated in Figure 2. The concentrations of the five metals tested were higher in roots than in shoots. The different biochar amendments reduced the concentrations of Pb, Zn, Cu, Cd and As in the shoot of plants. Compared with the CK group, adding biochar decreased the Pb, Zn, Cu, Cd and As contents in shoots by 63.9–89.5%, 46.9–66.0%, 32.7–62.4%, 40.4–76.4% and 54.9–77.5%, respectively. Among these biochar treatments, plants treated with HB3, SB0.4, MB1, SB0.4 and MB0.4 presented the lowest shoot Pb, Zn, Cu, Cd and As concentrations. However, there were no significant differences in the shoot metal concentrations of *Cassia alata* L. plants among different biochar addition rates for the same type of biochar. 

In general, plants treated with biochar increased the root Pb, Zn, Cu, Cd and As concentrations. Only the SB3 and HB0.4 treatments reduced the root Pb concentrations, by 17.3% and 15.5% respectively, but the results showed no significant differences. All biochar treatments increased the root Zn concentrations, by 1.05–1.30 times compared with the concentrations measured for the CK group. The MB treatments produced significant differences in root accumulation of Cu: MB0.4 and MB1 increased the concentration of Cu in roots by 37.2% and 24.2%, respectively, while the MB3 treatment decreased the concentration by 18.1%. None of the biochar treatments produced significant differences in root Cd content, while the SB3, MB3 and MB1 treatments reduced the root accumulation of Cd. HB0.4 and SB3 treatments reduced the uptake of As slightly, while the other treatments increased As differently.

### 3.3. Effects of Different Biochar Treatments on pH, TC, and TN in Mine Tailings

As shown in Table 3, the plant itself as well as the plant-biochar system could significantly increase the pH of mine tailings soil. The soil pH value of the control group was 6.5, which is faintly acidic, but the soil amendments increased this pH value to 6.8–7.1 due to the alkalinity of biochar and the application of large amounts of carbonates.

TC was affected by the rate of biochar addition as well as the biochar type. The TC contents of the HB treatments were significantly higher than those of the other biochar treatments. The TC contents of HB0.4, HB1 and HB3 were 159.24%, 355.13% and 590.01% higher than the TC content of CK, respectively. The TC content of SB3 was 56.01% higher than that of CK, while other treatments had no effect on TC content. Similar to the changes in TC contents, TN increased with the rate of biochar addition (Table 3). The TN contents of the HB0.4, HB1 and HB3 treatments were 120%, 90% and 100% higher than the TN content of CK, respectively. The TN contents of MB0.4, MB1 and MB3 were 20%, 40% and 140% higher than the TN content of CK, respectively. In addition, the TN contents of the SB treatments were significantly higher than those of the other biochar treatments: the TN contents of SB0.4, SB1 and SB3 were 80%, 210% and 650% higher than the TN content of CK. 

### 3.4. Effects of Different Biochar Treatments on NH_4_NO_3_-Extractable Metal Content in Mine Tailings

Table 4 shows that the mine tailings were naturally low in NH_4_NO_3_-extractable metal contents. In general, the biochar treatments did not affect the contents of NH_4_NO_3_-extractable Pb and Cu, while small increments in the contents of NH_4_NO_3_-extractable Zn, Cd and As were observed. HB1 increased the NH_4_NO_3_-extractable Zn and As contents by 35% and 30%, respectively. The NH_4_NO_3_-extractable Zn and Cd contents under the SB treatment were 1.2–1.3, 1.2–1.4 times those of CK, respectively. In addition, the NH_4_NO_3_-extractable As contents under the MB treatments were significantly higher than those of CK: the contents under the MB0.4, MB1 and MB3 treatments were 23.1%, 30.8% and 35.9% higher than the content of CK, respectively.

### 3.5. Effects of Different Biochar Treatments on Chemical Fractionation of Metals in Mine Tailings

**Figure 3:**
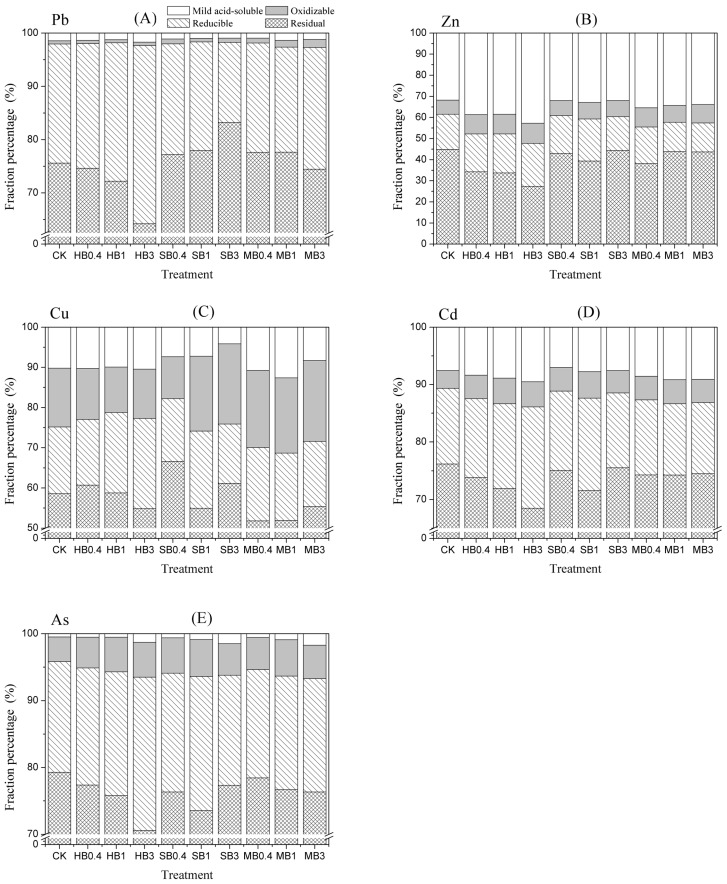
Effects of HB, SB and MB treatments on chemical fractionation of (**A**) Pb, (**B**) Zn, (**C**) Cu, (**D**) Cd and (**E**) As in mine tailings (*n* = 3). CK, HB/SB/MB0.4, HB/SB/MB1 and HB/SB/MB3 refer to no biochar input and the incorporation of biochar into the soil at 0.4%, 1% and 3% by mass, respectively. HB: Hibiscus cannabinus core biochar; SB: sewage sludge biochar; MB:chicken manure biochar.

Table 5 and Figure 3 show that the chemical fractionation of Pb, Cu, Cd and As in the mine tailings mainly existed in the residual fraction. The residual fractions in the mine tailing have been considered as inert and inaccessible to biota [20]. All biochar treatments produced low mild acid-soluble Pb and As contents, which indicated low mobility in the mine tailings. The addition of biochar significantly reduced the activities of Pb and Cu, with the SB treatments decreasing the activities of mild acid-soluble Pb and Cu by 23.4–34.5% and 27.8–59.1%, respectively. In addition, the mild acid-soluble Pb and Cu contents decreased with the rate of biochar addition. In contrast, a difference in the mild acid-soluble Zn, Cd and As contents was observed among the biochar treatments; the mild acid-soluble Zn, Cd and As contents of the HB treatments were 21.3–34.3%, 10.7–25.9% and 6.1–165.3% higher than those of CK, respectively. Most intriguingly, adding MB had the most significant effect on the mild acid-soluble As content, which was 16.4–250% higher than that of CK. Increasing the amount of added biochar stimulated the leaching of As, and the mild acid-soluble As contents of HB3 and SB3 were 2.6 and 3 times the content of CK.

## 4. Discussion

### 4.1. Effects of Biochar on Plant Growth and Heavy Metal Concentration

The main factors affecting metal tolerance in plants are growth speed, high biomass, heavy metal tolerance and low metal accumulation in shoots [21]. The results of our study show that *Cassia alata* L. could be a potential phytostabilization plant in high-multi-metal-contaminated soils, and biochar amendment can help increase the plant shoot biomass as well as the root biomass. In our study, all three biochars significantly increased the shoot and root biomass of plants. The SB3 treatment showed the best effect on plant growth in the growth substrate, increasing the biomass of shoots and roots of plants by factors of 2.8 and 3.2, respectively. These positive effects on the biomass of plants after biochar addition have been observed in research on corn and other plants [13,22]. This finding may be attributed to the reduction in metal toxicity caused by immobilization and biochar-provided nutrients [23]. Our research shows that biochar significantly increased the TC and TN contents, which might be attributed to biochar feedstocks and the high TC and TN contents of biochar. SB generally showed a higher TN content than the other biochars, enabling it to supply nutrients to enhance plant growth [24,25]. However, not all types of biochar showed improved performance at high application rates. For example, The HB0.4 and MB0.4 treatments produced higher biomass than the other rates of biochar addition. This finding is similar to that reported by Rillig et al. [26], who showed that plant biomass depended on biochar type and addition rate, with positive effects at low rate of biochar addition (<20%, *v*/*v*), but negative effects for higher rates. The reason is that some biochar can not only increase nutrient retention and increase nutrient efficiency but may also decrease the availability of nutrients by adsorbing phosphate in such poor nutrients as mine tailings [27,28]. In addition, high concentration of biochar addition may change soil function by inducing adverse impact on microbial community structure and activity [29], which may also affect the plant growth. Therefore, the suitable addition rate of biochar combined with other organic amendment might be suitable to regulate both nutrient and heavy metal stabilization in nutrient poor mine tailing.

Low metal accumulation in shoots is another important standard for assessing the phytostabilization potential of plants grown on multi-metal-contaminated soils, which would help control the spread of heavy metals to surrounding areas and reduce the long-term threat to ecosystems, food security and human health. Our results showed that *Cassia alata* L. did not accumulate high concentrations of heavy metals in shoots in general. In contrast, metals were precipitated or accumulated in roots. Himd et al. [30] indicated that biochar application could reduce Pb, Cd and Zn concentrations in the shoots of bean. Zheng et al. [31] also conducted an experiment involving the application of three biochars to a multi-metal-polluted soil with rice, and the biochars reduced the shoot metal concentration. However, our research showed no significant differences in the Pb, Zn, Cu, Cd and As concentrations of the shoots of plants, regardless of the biochar addition rate. This finding can be attributed to the immobilization of bioavailable metals as well as the dilution effect caused by increasing plant biomass [32]. In our study, application of biochar significantly increased the soil mild acid-soluble As, whilst the As content in shoot still decreased remarkably. This is in line with the results reported by Beesley et al. [33], in which the solubility and mobility of As were increased by orchard prune residue biochar amendment, but uptake to tomato plant was reduced. Such a phenomenon could be explained by that compartmentalisation of As in the roots of plant has been identified in As(III) spiked soils as root cell damage above toxic As thresholds can reduce transport of As upwards in the plant. The competition between As and other element linked with the mobility and bioavailability of As species, e.g. P originated from the sludge derived biochar [34], allowed As (V) to be more retained, which consequently led to the reduction of As uptake to plant [33]. In addition, the formation of soluble As-DOC (dissolved organic carbon) complexes might be another mechanism, since As in this form are mobile but might not be able to diffuse through the tissue of the plant [35].

### 4.2. Effect of Biochar on Mine Tailings

The mine tailings used in our study contained high multi-metal concentrations and low organic matter and nutrient contents, which limited natural plant establishment. In abandoned mine tailings ponds, biochar can be used to improve soil quality, reduce the mobility of heavy metals and increase the pH and cation exchange capacity (CEC) [12], which will help restore soil function and enhance pioneer plant establishment. The results of our research suggest that the pH of mine tailings increased with the addition rate of biochar, regardless of the biochar type (HB, SB and MB). This finding is in line with previous studies indicating that biochar pH and the liming effect could increase soil pH [12,13]. Reverchon et al. [36] also described increases in soil pH following biochar application to acidic soil. Increasing pH can decrease the mild acid-soluble Pb and Cu concentrations in mine tailings. Because HB, SB and MB are alkaline soil amendments, they can regulate soil pH. The pH of the SB treatment was 6.83, and the mild acid-soluble Pb and Cu contents still decreased significantly. This finding may be linked to the high ash content of the SB treatment [37].

Previous studies have indicated that the properties of biochar are conducive to metal stabilization. The high surface area, spatial structure and various organic functional groups of biochar could reduce mild acid-soluble metals [38,39]. HB is a low-ash biochar with a carbon content greater than 75%, while SB is a high-ash biochar with a carbon content less than 20%. The results showed that SB had a better effect than the HB treatment on metal immobilization. The results are similar to those of Yang et al. [34], whose soil incubation experiment showed that sewage sludge biochar had a better effect on Pb immobilization. The metal (Pb and Cu) sorption mechanism of biochar is attributed to metal precipitation, providing surface Ca^2+^ and Mg^2+^ with which to exchange metal and organic hydroxyl and carboxyl functional groups for surface complexation [40,41]. Cao et al. [42] noted that for SB containing a high P content, the dissolution of P is attributed to the presence of Pb and precipitation. Therefore, the reduction of mild acid-soluble Pb and Cu may further promote the growth of *Cassia alata* L. However, the biochars tested in this study had little positive effect on the immobilization of As, Zn and Cd. At a 3% addition rate in particular, the content of mild acid-soluble As increased significantly. An increase in arsenic availability has also been observed in other studies on soil and plant systems [43]. It has been reported that biochar amendment can increase the soil pH due to its alkaline effect, which may further increase As release. In our study, the pH of different biochar treatments significantly increased in the pot experiment. Increased soil pH will reduce positively charged sites in soil, hence the sites available for the As binding are reduced in the biochar treated soil [35]. Meanwhile, the biochar can act as electron donors to govern the rapid reduction of As(V) to As(III) through its O-containing functional groups. In addition, other studies [44,45] have indicated that the redox reaction of Fe, biotransformation of As and high P content of biochar are related to the enhanced concentration of mild acid-soluble As. Sewage sludge biochar as a soil amendment for acid soil remediation represents a potential application of abundant sewage sludge [41]. Compared with plant biochar, the SB treatment had a better effect on Pb and Cu immobilization. In addition, SB contains more nutrients, which may prevent nutrient leaching and increase plant biomass in multi-metal mine tailings. Although the pyrolyzed sewage sludge biochar showed better heavy metal stability, Zn and Cd, present in high concentrations, have high activities [46]. In our study, SB as a soil amendment was more suitable for *Cassia alata* L. plant establishment in multi-metal mine tailings. However, the potential risks of mild acid-soluble As release and As activation caused by SB in the high-As-contaminated mine tailings deserve further attention.

## 5. Conclusions

*Cassia alata* L. could be a potential phytostabilization plant in high-multi-metal-contaminated mine tailings because of its good growth and low heavy metal accumulation in shoots. Adding the three types of biochar examined in this study can increase plant growth and decrease heavy metal accumulation in shoots. The SB3 treatment showed the best effect on plant growth, increasing the biomass of shoots and roots, plant height and root length by factors of 2.8, 3.2, 1.5 and 3.3 compared with the CK treatment, respectively. The biochars can increase the soil pH, TC, and TN and decrease the mild acid-soluble Pb and Cu concentrations in the mine tailings. However, all of these biochars had little positive effect on the immobilization of As, Zn and Cd, and the potential risks of As release and activation observed with increasing biochar dose deserve further attention. Overall, *Cassia alata* L. plant establishment in such multi-metal mine tailings can be enhanced by biochar amendment, where the SB3 treatment might be the best choice, but the potential risk of As release must still be monitored and controlled.

## Figures and Tables

**Table 1:** Basic properties of mine tailings and different biochars.

Material	HB	SB	MB	MT	SEQS
Pb (mg·kg^−1^)	35	71.4	12.4	3642.7	500
Zn (mg·kg^−1^)	100.3	813.4	328	981	500
Cu (mg·kg^−1^)	0.2	203.5	40.4	70.5	400
Cd (mg·kg^−1^)	0.3	2.6	0.2	31.8	1
As (mg·kg^−1^)	1.8	18.7	3.4	1587.1	40
pH	7.5	7.1	9.1	6.5	-
C (%)	75.1	19.3	7	0.3	-
H (%)	2.9	1.3	0.5	0.3	-
N (%)	0.39	2.75	0.71	0.01	-
S (%)	0.21	0.35	0.47	1.3	-
BET Surface (m^2^·g^−1^)	117.61	14.10	6.52	-	-
Langmuir Surface (m^2^·g^−1^)	232.63	71.46	20.74	-	-
Micro-pore volume (cm^3^·g^−1^)	0.024	−0.00073	−0.0013	-	-
Median pore width (nm)	0.73	0.74	0.74	-	-

HB: *Hibiscus cannabinus* core biochar; SB: sewage sludge biochar; MB: chicken manure biochar; MT: Mine Tailing; SEQS: Soil environmental quality standard in China (GB 15618-1995).

**Table 2:** Experimental design for pot trial.

Group	Number	Mine Tailings (kg)	Amendment (*w*/*w*)	Repetition
Control group	CK	1	-	3
Amendment Group	HB0.4	1	0.4% HB	3
	HB1	1	1% HB	3
	HB3	1	3% HB	3
	SB0.4	1	0.4% SB	3
	SB1	1	1% SB	3
	SB3	1	3% SB	3
	MB0.4	1	0.4% MB	3
	MB1	1	1% MB	3
	MB3	1	3% MB	3

HB: *Hibiscus cannabinus* core biochar; SB: sewage sludge biochar; MB: chicken manure biochar. CK, HB/SB/MB0.4, HB/SB/MB1 and HB/SB/MB3 refer to no biochar input and the incorporation of biochar into the soil at 0.4%, 1% and 3% by mass, respectively.

**Table 3:** Effects of HB, SB and MB treatments on pH, TC, and TN in mine tailings.

Treatment	pH ^1^	TC ^1^ (g·kg^−1^)	TN ^1^ (g·kg^−1^)
CK	6.53 ± 0.02f	3.41 ± 0.07d	0.1 ± 0.01f
HB0.4	7.13 ± 0.01a	8.84 ± 0.46cd	0.22 ± 0.02c
HB1	7.05 ± 0.03b	15.52 ± 4.47b	0.19 ± 0.05cd
HB3	7.08 ± 0.04ab	23.7 ± 1.51a	0.2 ± 0.02cd
SB0.4	6.89 ± 0.01de	4.52 ± 0.2cd	0.18 ± 0.01cde
SB1	6.88 ± 0.03e	5.32 ± 0.12cd	0.31 ± 0.01b
SB3	6.83 ± 0.02e	8.28 ± 0.21c	0.75 ± 0.03a
MB0.4	7.03 ± 0.01b	4.03 ± 0.26cd	0.12 ± 0.01ef
MB1	7.01 ± 0.02bc	4.12 ± 0.08cd	0.14 ± 0def
MB3	6.95 ± 0.03cd	5.47 ± 0.04cd	0.24 ± 0.01c

^1^ Values represent the mean ± standard error (*n* = 3), different letters indicate significant differences among different treatments at a significance level of 0.05.

**Table 4:** Effects of HB, SB and MB treatments on NH_4_NO_3_-extractable Pb, Zn, Cu, Cd and As contents in mine tailings.

Treatment	NH_4_NO_3_-Extractable Metal Contents (mg·kg^−1^)				
	Pb ^1^	Zn ^1^	Cu ^1^	Cd ^1^	As ^1^
CK	0.35 ± 0.02ab	3.42 ± 0.1d	0.39 ± 0.00abc	0.1 ± 0.00de	0.39 ± 0.04cd
HB0.4	0.34 ± 0.01ab	3.17 ± 0.09d	0.39 ± 0.00abc	0.08 ± 0.00e	0.38 ± 0.02cd
HB1	0.35 ± 0.01ab	4.62 ± 0.33a	0.39 ± 0.01abc	0.13 ± 0.01ab	0.34 ± 0.01d
HB3	0.37 ± 0.01ab	4.17 ± 0.26abc	0.38 ± 0.00bcd	0.12 ± 0.01abc	0.43 ± 0.02abcd
SB0.4	0.34 ± 0.02ab	4.32 ± 0.17ab	0.37 ± 0.00cd	0.12 ± 0.01abcd	0.35 ± 0.01d
SB1	0.46 ± 0.11a	4.14 ± 0.1abc	0.38 ± 0.00cd	0.14 ± 0.01a	0.42 ± 0.04abcd
SB3	0.34 ± 0.00ab	4.32 ± 0.16ab	0.36 ± 0.00d	0.13 ± 0.01ab	0.41 ± 0.03bcd
MB0.4	0.35 ± 0.01ab	3.78 ± 0.14bcd	0.4 ± 0.02ab	0.1 ± 0.00de	0.48 ± 0.05abc
MB1	0.33 ± 0.00b	3.68 ± 0.16cd	0.4 ± 0.01ab	0.11 ± 0.01bcd	0.51 ± 0.06ab
MB3	0.36 ± 0.01ab	3.7 ± 0.22cd	0.41 ± 0.01a	0.1 ± 0.00cde	0.53 ± 0.02a

^1^ Values represent the mean ± standard error (*n* = 3), different letters indicate significant differences among different treatments at a significance level of 0.05.

**Table 5:** Effects of HB, SB and MB treatments on chemical fractionation of metal in mine tailings (%).

Metal	Treatment	Mild Acid-Soluble ^1^	Reducible Fraction ^1^	Oxidizable Fraction ^1^	Residual ^1^
Pb	CK	1.45 ± 0.26ab	22.34 ± 0.87b	0.61 ± 0.15b	75.6 ± 0.66a
	HB0.4	1.36 ± 0.1ab	23.38 ± 0.32ab	0.63 ± 0.1b	74.63 ± 0.18ab
	HB1	1.27 ± 0.18ab	25.99 ± 1.31ab	0.56 ± 0.08b	72.19 ± 1.53ab
	HB3	1.71 ± 0.12a	33.44 ± 1.37a	0.63 ± 0.26b	64.22 ± 1.38b
	SB0.4	1.11 ± 0.11ab	20.77 ± 6.47b	0.94 ± 0.14ab	77.19 ± 6.58a
	SB1	1.01 ± 0.11b	20.39 ± 1.79b	0.66 ± 0.05b	77.94 ± 1.69a
	SB3	0.95 ± 0.03b	15.02 ± 0.25b	0.82 ± 0.15ab	83.21 ± 0.4a
	MB0.4	0.96 ± 0.11b	20.53 ± 1.52b	0.9 ± 0.1ab	77.6 ± 1.73a
	MB1	1.38 ± 0.31ab	19.71 ± 1.56b	1.27 ± 0.29ab	77.64 ± 1.83a
	MB3	1.22 ± 0.29ab	22.83 ± 7.17b	1.51 ± 0.58a	74.45 ± 8ab
Zn	CK	31.81 ± 4.85a	16.65 ± 0.32a	6.69 ± 0.22c	44.85 ± 4.37a
	HB0.4	38.64 ± 1.95a	17.96 ± 0.58a	9.17 ± 1.12ab	34.24 ± 1.32abc
	HB1	41.31 ± 6.56a	18.71 ± 1.33a	9.61 ± 0.47a	30.37 ± 8.31bc
	HB3	42.72 ± 1.71a	20.42 ± 0.52a	9.56 ± 1.11a	27.3 ± 2.96c
	SB0.4	32.06 ± 1.38a	17.94 ± 5.63a	7.07 ± 0.3bc	42.93 ± 4.87ab
	SB1	32.89 ± 0.94a	19.92 ± 3.01a	7.84 ± 0.6abc	39.35 ± 1.8abc
	SB3	32.06 ± 1.82a	16.06 ± 0.85a	7.56 ± 0.3abc	44.32 ± 1.57a
	MB0.4	35.41 ± 3.22a	17.29 ± 0.37a	9.12 ± 0.17abc	38.18 ± 3.69abc
	MB1	34.32 ± 1.28a	13.89 ± 0.55a	7.97 ± 0.62abc	43.83 ± 1.34ab
	MB3	33.88 ± 3.4a	13.75 ± 0.31a	8.71 ± 0.82abc	43.66 ± 4.45ab
Cu	CK	10.2 ± 1.65ab	16.57 ± 0.25ab	14.64 ± 3.96a	58.59 ± 4.69a
	HB0.4	10.32 ± 0.68ab	16.34 ± 1.28ab	12.65 ± 2.3a	60.7 ± 3.03a
	HB1	9.93 ± 1.3ab	19.98 ± 0.97ab	11.31 ± 2.79a	58.78 ± 3.99a
	HB3	10.46 ± 0.14ab	22.39 ± 0.6a	12.29 ± 1.56a	54.86 ± 1.69a
	SB0.4	7.36 ± 0.71bc	15.59 ± 4.59b	10.45 ± 1.87a	66.59 ± 2.85a
	SB1	7.23 ± 0.72bc	19.15 ± 2.71ab	18.66 ± 2.28a	54.96 ± 3.77a
	SB3	4.17 ± 0.44c	14.71 ± 0.34b	19.98 ± 3.16a	61.15 ± 3.22a
	MB0.4	10.77 ± 1.1ab	18.2 ± 1.2ab	19.21 ± 2.94a	51.82 ± 5.22a
	MB1	12.61 ± 2.7a	16.71 ± 1.84ab	18.77 ± 5.12a	51.91 ± 9.49a
	MB3	8.32 ± 1.39ab	16.17 ± 1.34ab	20.16 ± 4.61a	55.35 ± 7.33a
Cd	CK	7.56 ± 1.21a	13.17 ± 0.37ab	3.1 ± 0.58a	76.18 ± 0.28a
	HB0.4	8.37 ± 0.34a	13.74 ± 0.15ab	4.08 ± 0.14a	73.8 ± 0.11ab
	HB1	8.88 ± 0.91a	14.73 ± 0.94ab	4.48 ± 0.4a	71.91 ± 0.96ab
	HB3	9.52 ± 0.24a	17.63 ± 0.41a	4.38 ± 0.48a	68.47 ± 1.12b
	SB0.4	7.03 ± 0.25a	13.8 ± 4.21ab	4.12 ± 0.58a	75.05 ± 4.35a
	SB1	7.75 ± 0.19a	16.05 ± 1.68ab	4.63 ± 0.42a	71.56 ± 1.57ab
	SB3	7.57 ± 0.4a	13.02 ± 0.3ab	3.9 ± 0.4a	75.51 ± 0.41a
	MB0.4	8.57 ± 1.02a	13.05 ± 0.99ab	4.11 ± 0.09a	74.27 ± 1.93ab
	MB1	9.16 ± 0.8a	12.39 ± 0.27b	4.21 ± 0.55a	74.25 ± 1.25ab
	MB3	9.1 ± 1.35a	12.37 ± 0.66b	4.04 ± 0.56a	74.49 ± 2.42ab
As	CK	0.49 ± 0.1d	16.55 ± 0.62a	3.69 ± 0.9a	79.28 ± 1.42a
	HB0.4	0.52 ± 0.05d	17.5 ± 0.51a	4.6 ± 0.38a	77.37 ± 0.4ab
	HB1	0.53 ± 0.09d	18.51 ± 1.5a	5.18 ± 0.54a	75.78 ± 0.91ab
	HB3	1.3 ± 0.2b	22.94 ± 0.69a	5.21 ± 0.56a	70.55 ± 1.42b
	SB0.4	0.63 ± 0.09cd	17.77 ± 5.41a	5.28 ± 0.93a	76.33 ± 5.94ab
	SB1	0.88 ± 0.06cd	20.05 ± 1.94a	5.53 ± 0.66a	73.55 ± 2.14ab
	SB3	1.49 ± 0.07ab	16.43 ± 0.33a	4.73 ± 0.51a	77.34 ± 0.71ab
	MB0.4	0.57 ± 0.06cd	16.2 ± 1.12a	4.78 ± 0.31a	78.44 ± 0.97ab
	MB1	0.92 ± 0.07c	16.97 ± 1.01a	5.43 ± 1.06a	76.68 ± 2.13ab
	MB3	1.73 ± 0.23a	16.94 ± 2.06a	4.99 ± 0.61a	76.33 ± 2.65ab

^1^ Values represent the mean ± standard error (*n* = 3), different letters indicate significant differences among different treatments at a significance level of 0.05.
